# Antagonistic potential of *Pseudomonasgraminis* 49M against *Erwiniaamylovora*, the causal agent of fire blight

**DOI:** 10.1007/s00203-016-1207-7

**Published:** 2016-03-22

**Authors:** Artur Mikiciński, Piotr Sobiczewski, Joanna Puławska, Eligio Malusa

**Affiliations:** Research Institute of Horticulture, Konstytucji 3 Maja 1/3, 96–100 Skierniewice, Poland

**Keywords:** Antagonistic bacteria, Siderophores, AHL, Biofilm, Antibiotic genes

## Abstract

In a previous study (Mikiciński et al. in Eur J Plant Pathol, doi:10.1007/s10658-015-0837-y, 2015), we described the characterization of novel strain 49M of *Pseudomonasgraminis*, isolated from the phyllosphere of apple trees in Poland showing a good protective activity against fire blight on different organs of host plants. We now report investigations to clarify the basis for this activity. Strain 49M was found to produce siderophores on a medium containing complex CAS-Fe^3+^ and HDTMA, but was not able to produce *N*-acyl homoserine lactones (AHLs). Moreover, it formed a biofilm on polystyrene and polyvinyl chloride (PVC) surfaces. Strain 49M gave a positive reaction in PCR with primers complementary to *gac*A, the regulatory gene influencing the production of several secondary metabolites including antibiotics. The genes *prn*D (encoding pyrrolnitrin), *plt*C, *plt*B (pyoluteorin), *phl*D (2,4-diacetyl-phloroglucinol) and *phz*C as well as *phz*D (and their homologs *phz*F and *phz*A encoding phenazine), described for antagonistic fluorescent pseudomonads, however, were not detected. Research into the biotic relationship between strain 49M and *Erwiniaamylovora* strain Ea659 on five microbiological media showed that this strain clearly inhibited the growth of the pathogen on King’s B and nutrient agar with glycerol media, to a very small extent on nutrient agar with sucrose, and not at all on Luria–Bertani agar. On medium 925, strain 49M even stimulated *E*. *amylovora* growth. The addition of ferric chloride to King’s B resulted in the loss of its inhibitory ability. Testing the survival of 49M in vitro showed its resistance to drought, greater than that of *E*. *amylovora*.

## Introduction

Fire blight (*Erwiniaamylovora*) is a very destructive bacterial disease of apple, pear and many other plant species, especially of the *Rosaceae* family (Vanneste 2000). Because of the harmfulness of the disease, its causal agent, the bacterium *E*. *amylovora*, has been placed in the list of EPPO and EU plant quarantine organisms, but only when occurring on nursery material (Directive 2000/29/EC of May, 8, 2000). For disease control, several methods have been proposed, including the use of antibiotics and copper-based preparations. Their efficacy depends, among others, on factors related to the amount of the inoculum, weather conditions and the susceptibility of protected plant tissues to fire blight. A very small assortment of measures for fire blight control and limitations related to the use of chemical method has stimulated the search for new methods and products. In recent years, biological control based on the use of beneficial and antagonistic bacteria and yeast has been developed (Johnson and Stockwell 2000; van der Zwet et al. 2012). Bacteria showing protective activity against fire blight are often present among the microorganisms inhabiting various natural environments, including plants (Mikiciński et al. 2008; Kabeil et al. 2010; Gerami et al. 2013; Roselló et al. 2013).

Several mechanisms have been proposed to explain the inhibitory effect of different bacterial antagonists on *E*. *amylovora*, including the production of toxic secondary metabolites and competition for nutrients and space (Beer and Rundle 1983; Wilson et al. 1992; Wilson and Lindow 1993; Stockwell et al. 1999; Cabrefiga et al. 2007; Paternoster et al. 2010). It seems that the action of the most efficient biocontrol agents, e.g., *Pantoeaagglomerans* (syn. *Pantoeavagans*) strains C9-1, Eh252, Eh1087 and Eh318, against fire blight is based on antibiosis (Ishimaru et al. 1988; Davis and Ishimaru 1993; Kearns and Hale 1993; Wright et al. 2001; Stockwell et al. 2002). In the case of *Pseudomonasfluorescens* strain A506, however, it was documented that the suppression of the pathogen relies mainly on competing for sites and nutrients required for the growth of *E*. *amylovora* (Wilson and Lindow 1993; Stockwell et al. 1999; Johnson and Stockwell 2000; Temple et al. 2004). Also, the effective strain EPS62e of *P*. *fluorescens*, isolated in Spain, does not produce antimicrobial compounds, but acts by cell-to-cell interference as well as by differences in growth potential and in the efficiency of nutrient use (Cabrefiga et al. 2007). On the other hand, there are examples that pseudomonads are capable of producing a broad spectrum of antibiotics (De Souza and Raaijmakers 2003; Delaney et al. 2001; McSpadden-Gardener et al. 2001; Mavrodi et al. 2001). Recently, a novel approach for fire blight control using lactic acid bacteria was demonstrated. *Lactobacillusplantarum* strains PC40, PM411, TC54 and TC92 were effective in the protection of detached apple and pear organs against disease. One of the mechanisms responsible for their mode of action is based on bacteriocin production, indicated by the presence of several genes involved in their biosynthesis (Roselló et al. 2013).

One of the important features of a potential of biocontrol agent is the ability to produce siderophores, i.e., ferric-specific ligands that solubilize extracellular iron (III) and are transported by specific outer membrane receptors into the bacterial cell (Neilands 1984). Bacteria with the ability to produce siderophores may compete with other microorganisms for the iron ions that are necessary to basic life processes (Höfte et al. 1992). Siderophores produced by pseudomonads such as pyoverdine can also act as antibiotics (Kraus and Loper 1992, 1995).

Another important feature of bacteria colonizing the same biotope is communication via mediators in a process called quorum sensing (QS). Bacteria often secrete small compounds, such as *N*-acyl homoserine lactones (AHLs) (McClean et al. 1997; von Bodman et al. 2003; Jakovljevic et al. 2008) or autoinducer 2 (Al-2), a furanosyl diborate or oligopeptides present in some Gram-negative or positive bacteria, respectively (Federle and Bassler 2003). It was proved that AHL production influences the efficacy of bacterial biocontrol agents (Jakovljevic et al. 2008). Bacteria have also developed some other strategies to be competitive with other microorganisms, e.g., the formation of a biofilm, which is a structure composed of bacterial cells suspended in a mass of extracellular polymers (secreted in the form of an ooze), which assists in adhesion to various surfaces (Hossain and Tsuyumu 2006).

In our previous work, strain 49M of *Pseudomonasgraminis* was shown to have good protective action against fire blight under laboratory and greenhouse conditions (Mikiciński et al. 2008; 2015; unpublished data). The purpose of the present work was to investigate the putative mechanisms related to this protective activity of the novel strain 49M. To this end, the biotic relationships between this strain and *E*. *amylovora*, the presence of genes encoding for the synthesis of some antibiotics, the production of siderophores and acylated homoserine lactones (AHLs), the ability to form a biofilm and the resistance to desiccation were studied.

## Materials and methods

### Bacterial strains

Strain 49M of *P*. *graminis*, isolated from an apple phyllosphere in the Pomological Orchard in Skierniewice, Poland, in 2006 and strain Ea659 of *E*. *amylovora* isolated from an apple terminal shoot in Poland, were used in this study. The following reference strains were included: A506 (*P*. *fluorescens*), C9-1 (*P*. *vagans*), 30-84 (*P*. *chlororaphis* (*aureofaciens*)), Pf5 (*P*. *fluorescens*) and CV026 *Chromobacterium violaceum*.

### Biotic relations on agar media

The ability of strain 49M to inhibit the growth of *E*. *amylovora* was determined on five agar media: nutrient agar sucrose (NAS), LB (tryptone 1 %, NaCl 1 %, yeast extract 0.5 %, agar 2 %), King’s B (KB), NAG (2.3 % Difco nutrient agar, 1.6 % glycerol) (Schaad et al. 2001) and 925 medium (glucose, 0.5 %, K_2_HPO_4_ 0.3 %, NaH_2_PO_4_ 0.1 %, NH_4_Cl 0.1 %, MgSO_4_·7H_2_O 0.03 %, agar 1.5 %) (Kado 1979). The test was also performed on KB medium with the addition of 50 µM of iron chloride and on 925 medium with the addition of iron citrate in amounts of 0.001, 0.5 and 1 mM. Bacteria were seeded on each medium in the center of Petri dishes, and after 3 days of incubation at 26 °C, they were killed by chloroform vapor and then flooded with 4 ml of melted soft agar mixed with 0.1 ml of strain Ea659 suspension (1 × 10^8^ CFU·ml^−1^), according to Vidaver et al. (1972) with slight modifications. The radius of growth inhibition or stimulation of the pathogen (in the case of 925 medium) was measured after 24 and 48 h. Tests on each medium were carried out in three replicates (Petri dishes). Strains A506 and C9-1 were used for comparison.

### Production of siderophores (CAS agar plates)

The test was performed on medium containing a complex of CAS-Fe^3+^-HDTMA, prepared according to Schwyn and Neilands (1987). Chrome azurol S (CAS) was dissolved in water and mixed with FeCl_3_·6H_2_O and HCl. The solution was slowly added to a water solution of hexadecyltrimethylammonium (HDTMA) and autoclaved. A mixture of MM9 salts (derived from M9 medium by the reduction of phosphate to 0.03 % KH_2_PO_4_), agar, Pipes buffer (pH 6.8) and NaOH solution was prepared separately, and after autoclaving and cooling, the casamino acid and carbon source (glucose) were added. The solutions were mixed without the generation of foam. Bacteria were spot-seeded on the surface of the medium and incubated at 26 °C for 48 h. A change of color from blue to red-orange around bacterial growth indicated siderophore production. Strains A506 and C9-1 were used for comparison.

### Production of *N*-acyl homoserine lactones

Production of *N*-acyl homoserine lactones (AHLs) by bacteria was tested in the presence of the AHL indicator strain *Chromobacteriumviolaceum* CV026 according to the method of McClean et al. (1997). CV026 was line-streaked on solidified LB medium. Perpendicularly, the tested strains were also streaked at a distance of about 3 mm from the CV026 line. Production of the signaling molecule AHL by the tested strains was indicated by violet pigmentation of CV026. Strains A506 and C9-1 were used for comparison.

### Biofilm formation

Bacteria were grown overnight in YP medium (1 % peptone, 0.5 % yeast extract) and then were diluted ten times with the same medium or LB. The suspension was added to each well of a 96-well microtiter plate made from polystyrene or polyvinyl chloride (PVC) and incubated for 12 h at 27 °C according to the method of Hossain and Tsuyumu (2006). Subsequently, the plates were rinsed repeatedly and thoroughly with distilled water. Sedentary bacteria were quantified by the addition of 125 µl of 1 % crystal violet (CV) solution and incubation for 15 min. CV-stained biofilms were rinsed with sterile distilled water and solubilized in 200 µl of 95 % ethanol for 10–15 min at room temperature. The ethanol extracts from ten wells were transferred to the tube for a total volume of 2 ml. The absorbance of this solution was determined by a spectrophotometer (Helios β) at 600 nm. Strains A506 and C9-1 were included for comparison. The experiment was performed in three replicates.

### Detection of genes encoding for antibiotic production

The ability of 49M to produce antibiotics was determined by PCR with primers complementary to the following genes: *prnD* (pyrrolnitrin), *pltC*, *pltB* (pyoluteorin), *phlD* (2,4-diacetylphloroglucinol) and *phzC*, *phzD* (phenazine) homologous of *phzF* and *phzA*, respectively, and *gacA* (a regulatory gene) (Table 1). As a template, 1 µl of boiled bacterial suspension (1 colony in 0.5 ml sterile H_2_O) was used for all PCRs. Reference strains 30-84 (*P*. *chlororaphis* (*aureofaciens*)) and Pf5 (*P*. *fluorescens*) were used as the positive control. Strain A506 was also included.

**Table 1:** Primers and amplification conditions for the different PCR-based screenings of genes encoding antibiotics

Antibiotic and genes	Primer sequence	Product bp	Amplification conditions	References
Pyrrolnitrin *prn*D	PRND1 (GGGGCGGGCCGTGGTGATGGA) PRND2 (YCCCGCSGCCTGYCTGGTCTG)	786	Initial denaturation at 95 °C for 2 min: 30 cycles of 95 °C for 60 s, 68 °C for 60 s and 72 °C for 60 s, final extension at 72 °C for 10 min	De Souza and Raaijmakers (2003)
Phenazine *phz*A, *phz*F or *phz*C, *phz*D	PHZ1 (GGCGACATGGTCAACGG) PHZ2 (CGGCTGGCGGCGTATTC)	1400	Initial denaturation at 94 °C for 2 min: 25 cycles of 94 °C for 60 s, 56 °C for 45 s and 72 °C for 105 s, final extension at 72 °C for 10 min	Delaney et al. (2001)
Pyoluteorin *plt*C	PLTC1 (AACAGATCGCCCCGGTACAGAACG) PLTC2 (AGGCCCGGACACTCAAGAAACTCG)	438	Initial denaturation at 95 °C for 2 min: 30 cycles of 95 °C for 60 s, 68 °C for 60 s and 72 °C for 60 s, final extension at 72 °C for 10 min	De Souza and Raaijmakers (2003)
*plt*B	PltBf (CGGAGCATGGACCCCCAGC) PltBr (GTGCCCGATATTGGTCTTGACCGAG)	800–900	Initial denaturation at 95 °C for 2 min: 30 cycles of 94 °C for 60 s, 58 °C for 45 s and 72 °C for 105 s, final extension at 72 °C for 10 min	Mavrodi et al. (2001)
2,4-Diacetyl-phloroglucinol *phl*D	B2BF (ACCCACCGCAGCATCGTTTATGAGC) BPR4 (CCGCCGGTATGGAAGATGAAAAAGTC) BPF3 (ACTTGATCAATGACCTGGGCCTGC)	600	Initial denaturation at 95 °C for 3 min: 34 cycles of 95 °C for 60 s, 60 °C for 60 s and 72 °C for 60 s, final extension at 72 °C for 10 min	McSpadden-Gardener et al. (2001)
Regulatory gene *gac*A	Gaca1 (GBATCGGMGGYCTBGARGC) Gaca2 (MGYCARYTCVACRTCTCTGSTGAT)	425	Initial denaturation at 95 °C for 2 min: 30 cycles of 95 °C for 60 s, 61 °C for 60 s and 72 °C for 60 s, final extension at 72 °C for 10 min	De Souza et al. (2003)

**Table 2:** Inhibition zone of *Erwinia* *amylovora* by the antagonistic bacterial strains on different media

Strain	Medium									
	NAS		LB		King’s B		NAG		925	
	24 h	48 h	24 h	48 h	24 h	48 h	24 h	48 h	24 h	48 h
49M	5#*	0	0	0	13.6 ± 0.3c	14.3 ± 0.3b	3.0 ± 0.0b	2.8 ± 0.2b	+18.0 ± 0.0c	+12.5 ± 0.3c
A506	0	0	0	0	11.5 ± 0.3b	0.0 ± 0.0a	6.0 ± 0.0c	5.6 ± 0.0c	+10.5 ± 0.3b	+10.0 ± 0.6b
C9-1	0	0	0	0	0.0 ± 0.0a	0.0 ± 0.0a	2.5 ± 0.3a	1.8 ± 0.3a	+4.3 ± 0.3a	+4.3 ± 0.3a

* Radius from the margin of the colony to margin of the inhibition zone (mm); # very weak, hazy zone; + zone of Ea659 growth stimulation; means within column followed by the same letter are not significantly different at *P* < 0.05 according to Newman–Keuls test (mean ± SE)

### Assessment of the survival of strain 49M (survival under dry in vitro conditions)

Fifty microliters of a suspension of strain 49M in sterile water (10^8^ CFU ml^−1^) was deposited on the surface of sterile microscope cover glasses placed in a closed Petri dish. They were kept at room temperature for 2–3 h to dry up and then incubated at 28 °C and 30 % relatively humidity (RH). The number of bacteria on the microscope glasses was determined for a period of 49 days at weekly intervals by washing the cover glasses in sterile distilled water, followed by serial dilution and seeding on NAS medium. In each period, a separate set of five cover glasses was analyzed. Strain Ea659 of *E*. *amylovora* was included for comparison.

**Fig. 1:**
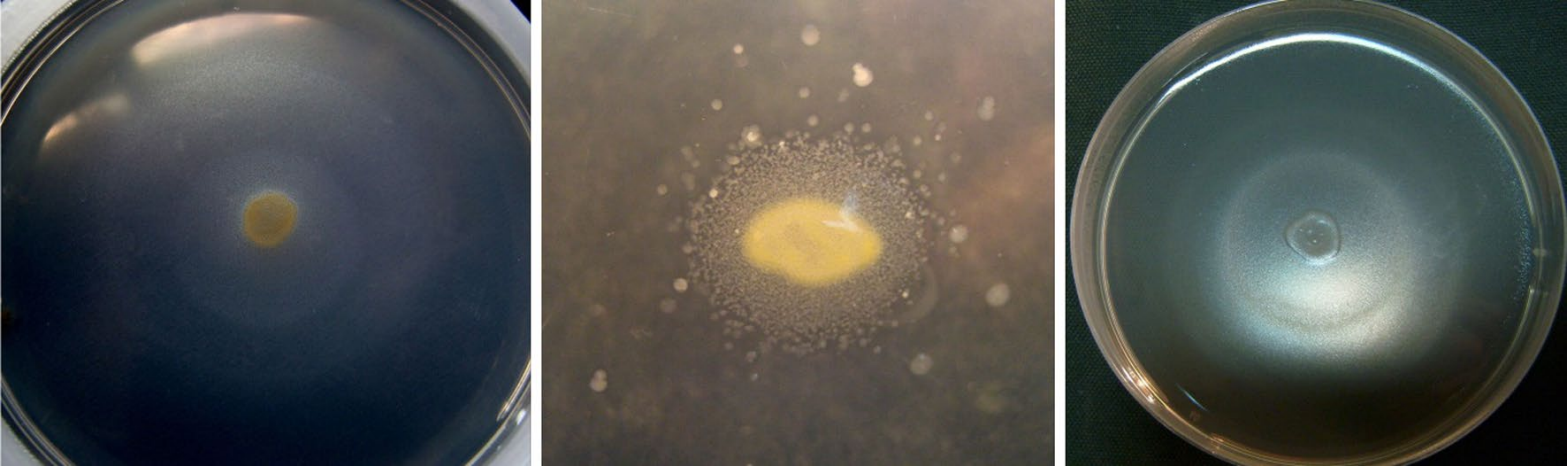
The growth stimulation zone of *Erwinia* *amylovora* on 925 medium supplemented with glucose around bacterial strains: from *left* 49M, C9-1, A506 (3 days after incubation)

**Table 3:** Production of siderophores and signaling molecules AHL by the antagonistic bacterial strains

Strain	Siderophores	AHL
C9-1	+	+
A506	+	−
49M	+	−

+, Positive; −, negative

### Statistical analysis

Results were subjected to one-way ANOVA using Newman–Keuls test for the separation of means at *P* ≤ 0.05. All calculations were done using STATISTICA software, version 10.

## Results

Our study on the biotic relationships between strain 49M and *E*. *amylovora* on five artificial media showed that the strain caused growth inhibition of the pathogen on KB, NAG and NAS, but at different rates. The largest inhibition zone occurred after 48 h of incubation on KB, while this was negligible on NAS. Strain A506 inhibited *E*. *amylovora* on KB and NAG; however, its activity on KB disappeared after 24 h. Strain C9-1 showed very low antagonistic activity only on NAG medium. The bacteria of all studied strains stimulated the growth of *E*. *amylovora* on 925 medium (Table 2; Fig. 1). The zone of stimulation was white-gray colored. After 24 h, in the case of strain 49M, the radius of the growth stimulation zone was the widest (18 mm) and its margin (2 mm) was even more strongly marked, which may indicate a more intensive growth of bacteria. After 48 h, the margin was broadened. The smallest growth stimulation zone occurred in the case of strain C9-1. The addition of 1 mM iron citrate to 925 medium resulted in a slight decrease in growth stimulation for A506 and C9-1 (data not shown). It should be pointed out, however, that *E*. *amylovora* did not grow on this medium when any of other bacteria were seeded on it. In the case of A506, the addition of iron citrate to this medium at all concentrations caused a loss of fluorescent pigment production by this strain. Also, the addition of iron chloride to KB medium resulted in the loss of inhibitory activity by strains 49M and A506 (data not shown).

**Fig. 2:**
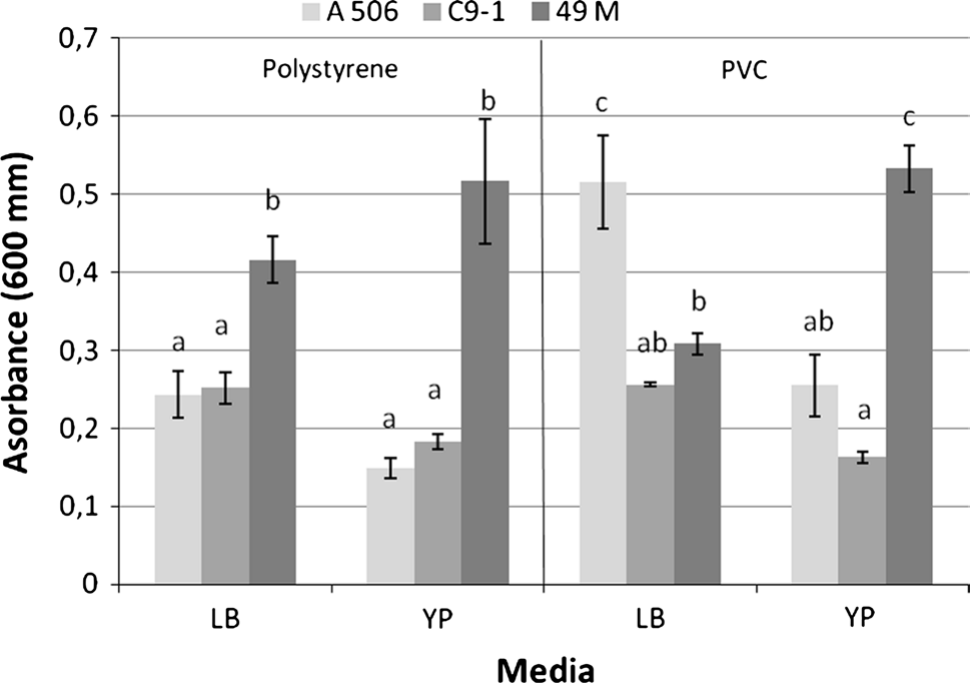
Attachment of 49M, A506 and C9-1 cells to polystyrene and polyvinyl chloride (PVC) well surfaces after cultivation in LB or YP liquid media for 12 h. Standard errors are indicated. Means within each medium and well material followed by the *same* *letter* are not significantly different at *P* < 0.05 according to Newman–Keuls test

Strain 49M produced siderophores, but not *N*-acyl homoserine lactones. Similar activities have been shown by strain A506. However, C9-1 produced both compounds (Table 3). A biofilm was formed by all studied strains on both polystyrene and PVC well surfaces. The highest value of absorbance (biofilm production) was shown by strain 49M on the polystyrene surface with both LB and YP liquid media (Fig. 2). A similar tendency was found on the PVC surface, but only when 49M was cultivated on YP medium; on LB, the absorbance was significantly lower than A506, but higher than C9-1.

No genes encoding for different antibiotics (*prnD*, *pltC*, *pltB*, *phlD* as well as *phzC*, *phzD* and their homologues *phzF* and *phzA*) were detected in the DNA of strain 49M (Table 4). However, the regulatory gene *gac*A, involved in the production of several secondary metabolites including bacteriocins, was found in this strain. As expected, the presence of these genes was confirmed in reference strains 30-84 of *P*. *chlororaphis* and Pf5 of *P*. *fluorescens*, respectively, included in the analysis for comparison.

**Table 4:** Presence of genes encoding antibiotics in the antagonistic bacterial strains

Strain	*Prn*D	*Phz*A, *phz*F, *phzC*, *phzD*	*Plt*C, *plt*B	*Phl*D	*Gac*A
A506	−	− − − −	− −	−	−
49M	−	− − − −	− −	−	+
*P*. *chlororaphis* 30-84	−	+ + + +	− −	−	Nd
*P*. *fluorescens* Pf5	+	− − − −	+ +	+	+

Nd, not done; +, positive; −, negative

In the survival test under dry conditions in vitro (30 % RH), living cells of strain 49M were detected during the entire experimental period (49 days), while those of Ea659 survived only 42 days (Table 5). The greatest decrease in bacterial cell number was recorded during the first 14 days, followed by a much slower gradual decrease in living cells.

**Table 5:** Survival of bacterial cells on sterile microscope cover glass surface (resistance to desiccation)

Strain	Number of bacteria (CFU) after days:						
	0	14	21	28	35	42	49
Ea659	5.0 × 10^6^	337.0 ± 45.3a	190.2 ± 33.4a	75.8 ± 20.8a	6.0 ± 1.5a	1.4 ± 1.2a	0.0 ± 0.0a
49M	5.0 × 10^6^	537.2 ± 55.5b	125.2 ± 41.0a	140.2 ± 52.8a	29.2 ± 7.4b	1.8 ± 0.9a	4.6 ± 1.5b

Analysis was made separately for each day; means within column followed by the same letter are not significantly different at *P* < 0.05 according to Newman–Keuls test (mean ± SE)

## Discussion

Of the several studied putative mechanisms for the protective activity against fire blight of *P*. *graminis* strain 49M, we found that it inhibited *E*. *amylovora* growth on three microbiological media, but at the highest rate on KB. This feature could be related to siderophore production on this medium. The role of these compounds in the control of some plant bacterial diseases has been described, e.g., *P*. *fluorescens* through siderophore-mediated competition for Fe^+3^ reduces the development of *Xanthomonasaxonopodis* pv. *malvacearum*, a pathogen of cotton (Mondal et al. 2000). Some evidence that siderophore production may be related to biocontrol was also obtained in the case of strain *P*. *fluorescens* F113 in the control of potato soft rot (*Erwiniacarotovora*, syn. *Pectobacteriumcarotovorum*) under iron-limiting conditions (Cronin et al. 1997). However, more recently, Temple et al. (2004) found that strain A506 of *P*. *fluorescens* produces an antibiotic inhibitory to *E*. *amylovora* on iron-amended medium 925, but the production of the siderophore pyoverdine was blocked on that medium. The authors believe that without information on the structure of antibiotic produced by A506 or the genes involved in its biosynthesis, this inverse relationship between pyoverdine production and antibiosis should be considered correlative, not causative. Simultaneously, it was also documented that the bioavailable iron concentrations on pome fruit flowers are too low to induce the expression of antibiosis (Temple et al. 2004). In the present work, the addition of iron chloride to KB medium caused the loss of inhibitory activity of both strains 49M and A506. Similar observations were found in a study by Cabrefiga et al. (2007) who showed that strain EPS62e of *P*. *fluorescens* developed antagonism against *E*. *amylovora* only on KB medium without the addition of iron in the form of iron chloride. On the other hand, we confirmed the production of siderophores by 49M and A506 on Schwyn–Neilands medium. However, it is difficult to assess the direct role of siderophores in the protective activity of both strains *inplanta*. Ülke and Ҫınar (1999) found that bacterial isolates causing the largest inhibition zones on KB medium appeared to be not the best protectants of pear fruitlets. Also, Beer et al. (1984) noted that, very often, bacteria that suppress the growth of *E*. *amylovora* in vitro are not able to activate this mechanism on plants. One of our earlier studies showed that isolates which did not have the ability to inhibit pathogen growth on KB or NAS media very effectively protected apple blossoms against fire blight (Mikiciński et al. 2008). Benlioğlu and Erdoğan (1999) reported that Gram-positive bacteria, fluorescent pseudomonads and yellow bacteria colonizing the apple, pear and quince phyllosphere exhibit the ability to produce antibiotics that can inhibit the growth of *E*. *amylovora*. However, Ülke and Ҫınar (1999) showed that of all bacteria obtained from different apple and pear organs, only 11.2 % negatively affected the growth of this pathogen on KB. Certainly, the composition of the growth medium affects the biotic relationships between bacteria co-cultivated on it, and the results may not be unquestioningly transferred to natural conditions.

In this context, it is noteworthy that all strains used in our study stimulated *E*. *amylovora* growth on 925 medium. It is thought that those bacteria utilized some compounds of this medium, at least glucose, and processed them into beneficial substances for the growth and multiplication of the pathogen. However, it should be pointed out that, in the above-mentioned study by Temple et al. (2004), 925 medium was supplemented with 1.5 % potassium gluconate (C_6_H_11_KO_7_) as the carbon source to study the antibiosis of strain A506 under different levels of iron. Instead of potassium gluconate, we used glucose (C_6_H_12_O_6_). There are no literature data on the reason for the observed differences, i.e., inhibition or stimulation of *E*. *amylovora* growth depending on the carbon source in this medium. Without additional detailed study, it is difficult to elucidate this unambiguously.

Our study shows that strain 49M, similar to A506, does not possess the main genes encoding for antibiotics described for fluorescent pseudomonads. However, in the genome of 49M, in contrast to A506, the regulatory gene *gacA* was found, which could be related to its broader spectrum of antagonistic activity to *E*. *amylovora*. Our findings clearly support the paradigm that preliminary selection of bacteria for biocontrol purposes based on in vitro tests is not sufficiently reliable. For the selection of effective strains against fire blight, Hevesi and El-Arabi (1999) developed a laboratory method using apple leaf disks. We show, in this respect, that screening on pear fruitlets or, if possible, on apple blossoms is also very useful.

Strain 49M did not produce acyl homoserine lactones (AHLs) involved in the cell–cell communications of many bacteria. This suggests that the quorum-sensing system (QS) based on AHL molecules does not play a role in the regulation of strain 49M metabolism. In some species of the genus *Pseudomonas*, however, such regulation has been shown (Venturi 2006). In the context of biocontrol, a good example is *P*. *chlororaphis* strain PA23 in which QS based on AHLs is involved in the production of secondary metabolites such as pyrrolnitrin and phenazine as well as biofilm formation (Selin et al. 2012). All three strains used in our study formed a biofilm, but only C9-1 produced AHLs, which suggests another mechanism responsible for this ability for strains 49M and A506. The significantly higher amount of biofilm formed by strain 49M in comparison with the other strains indicates the presence of a very important trait supporting its biocontrol potential activity. Bacterial biofilms are composed mainly of exopolysaccharide (EPS), proteins, lipids and nucleic acids (Davey and O’toole 2000). They protect bacteria against unfavorable environmental conditions such as UV radiation, pH shifts, osmotic shock or desiccation (Flemming 1993). Such conditions often occur on plant surfaces and are regarded as one of the main limiting factors for effective bacterial colonization. Thus, the production of a biofilm by an antagonist used for biocontrol can effectively increase its survival and activity and also contribute to maintaining an important source of water (Zhang et al. 1998). The good colonization and survival rate of strain 49M determined on apple flowers in an orchard (data not published) and its survival under dry in vitro conditions (30 % RH) could also be due to biofilm formation.

In conclusion, the findings of this study provide evidence that strain 49M of *P*. *graminis* produces toxic secondary metabolites, but not those encoded for by common antibiotic genes described for fluorescent pseudomonads. It also produces siderophores and forms a biofilm, but does not produce AHL signaling molecules, which means that a genetic background other than QS based on AHLs is responsible for those phenomena. It seems that at least some of these features are related to 49M protective activity against fire blight. Haas and Keel (2003) studied antibiotic production by root-colonizing pseudomonads and concluded that the expression of features important in biocontrol is subject to the complex process of regulation. On the functioning of this regulation, various factors such as the availability of nutrients (Duffy and Défago 1999; Michelsen and Stougaard 2012), the phase of bacteria growth, temperature, size of the population (Haas and Défago 2005) and also the presence of other microorganisms (Garbeva et al. 2011) could have an influence. Therefore, other mechanisms of antagonism such as competition for nutrients and space should also be considered. Future studies will focus on the biological capabilities of strain 49M and its persistence in the environment.
